# On-chip plasmonic waveguide optical waveplate

**DOI:** 10.1038/srep15794

**Published:** 2015-10-28

**Authors:** Linfei Gao, Yijie Huo, Kai Zang, Seonghyun Paik, Yusi Chen, James S. Harris, Zhiping Zhou

**Affiliations:** 1 State Key Laboratory of Advanced Optical Communication Systems and Networks, Peking University, Beijing 100871, China; 2 Department of Electrical Engineering, Stanford University, Stanford, CA 94305, USA

## Abstract

Polarization manipulation is essential in almost every photonic system ranging from telecommunications to bio-sensing to quantum information. This is traditionally achieved using bulk waveplates. With the developing trend of photonic systems towards integration and miniaturization, the need for an on-chip waveguide type waveplate becomes extremely urgent. However, this is very challenging using conventional dielectric waveguides, which usually require complex 3D geometries to alter the waveguide symmetry and are also difficult to create an arbitrary optical axis. Recently, a waveguide waveplate was realized using femtosecond laser writing, but the device length is in millimeter range. Here, for the first time we propose and experimentally demonstrate an ultracompact, on-chip waveplate using an asymmetric hybrid plasmonic waveguide to create an arbitrary optical axis. The device is only in several microns length and produced in a flexible integratable IC compatible format, thus opening up the potential for integration into a broad range of systems.

Polarization manipulation for photonic integrated circuits (PICs) has always been a significant concern because waveguides and devices are usually strongly polarization-dependent. Integrated polarization rotators converting between TE and TM modes have been proposed^1^,^2^,^3^. However, only TE and TM states are far from sufficient for many optoelectronic applications. For example, 0°/90°,  ± 45° linear polarization or circular polarization are needed to encode quantum information^4^,^5^,^6^,^7^, and circular polarization is usually a must in and spectroscopy^8^ and sensing^9^. Traditionally these applications are realized in free space using bulk optical waveplates for polarization manipulation. Because integration provides irreplaceable stability, scalability and cost reduction, these fields are experiencing an overwhelming trend towards integration^10^,^11^, thus making polarization rotation devices, like waveplates, but on-chip, are urgently needed for continued integration.

Traditionally, passive on-chip TE/TM rotators are achieved by dielectric waveguides. Their fundamental operating principles can be categorized into two main groups: mode evolution^12^,^13^ and mode beating^14^,^15^. Mode evolution schemes usually require quite large lengths (e.g. hundreds of microns) to achieve adiabatic mode change. Moreover, other polarization states beyond TE/TM, such as circular or linear polarization in a specified angle are difficult or impossible to realize by mode evolution. Mode beating is the principle upon which bulk waveplates based, but the key point in waveguide type devices is to alter the optical axis of the propagating eigenmode. Previous work utilized complicated tuned geometries, such as slanted surfaces^14^ or multi trenches^15^, and it is very hard to realize an arbitrary optical axis with conventional lithography process. Most recently, an advanced work reported realization of a rotated waveplate using femtosecond laser writing^16^, but the device length is 18 mm, and the fabrication is extremely complex and not available for easy incorporation into large-scale chip fabrication.

Meanwhile, plasmonics does show great potential for compact on-chip polarization manipulation devices^17^,^18^,^19^,^20^,^21^,^22^, because of the strong confinement and natural polarization sensitivity^22^,^23^,^24^,^25^,^26^,^27^,^28^. Compact TE/TM polarization rotators have been proposed^19^,^20^,^21^, however, most of these devices were based on the mode evolution principle. Plasmonic quarter waveplate have been reported^29^,^30^, but not in waveguide type configuration that could be incorporated into an integrated photonic circuit.

In this letter, for the first time, we propose and experimentally demonstrate the concept of an on-chip waveplate using an asymmetric hybrid plasmonic waveguide, to realize the full range of polarization states covering the entire Poincaré sphere. The structure is very simple and in a waveguide format that can be easily integrated into any photonic system wherever polarization rotation is needed. Moreover, it shows an outstanding performance, for example the polarization conversion efficiency from TE to TM as high as 99.2%.

## Results

### Structure and principle

**Figure 1:**
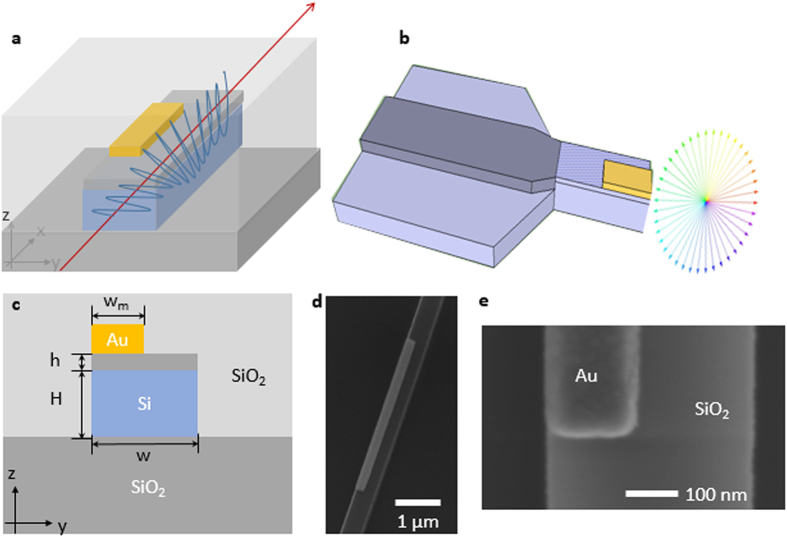
On-chip waveplate design and fabrication. (**a**) 3D view of the polarization rotating device integrated in-line with a standard Si wire waveguide of 400 nm width and 250 nm height. (**b**) Polarization rotation segment integrated at the output end of a laser to generate circular polarization. (**c**) Cross section of the polarization rotation device. It is an asymmetric hybrid plasmonic waveguide, consisting of a Si wire waveguide, a thin SiO_2_ gap and a partially covering metal strip on top. (**d**) Scanning electron micrograph (SEM) showing the device in-line integrated with a Si wire waveguide. (**e**) Top view SEM of the rotation section.

The design of our structure is described in Fig. 1. The device is based on standard SOI substrates. As shown in Fig. 1(c), the rotation section is a normal Si wire waveguide with a thin SiO_2_ gap and a partially covering metal strip on top, creating an asymmetry hybrid plasmonic waveguide (HPW). The rotation section can be flexibly introduced into a waveguide type section anywhere in a system or as part of a device. Figure 1(a) shows an example serving as a linear polarization rotator for an integrated system, and Fig. 1(b) illustrates how the device can be integrated as part of a laser to produce a designed output polarization, such as circular or other states, thus overcoming the limitation in lasers that only TE or TM polarization can be generated due to the properties of active materials^31^,^32^.

**Figure 2:**
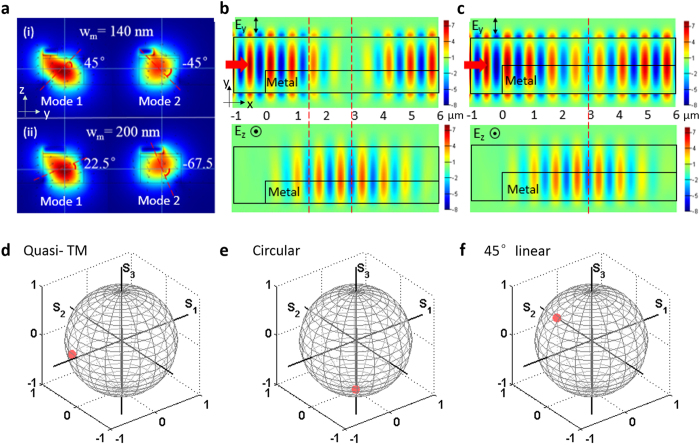
Simulation. (**a**) Magnetic field profiles of the two eigenmodes when θ of the first eigenmode is 45°(**i**) and 22.5° (**ii**), respectively. Arrows show the electric field direction. (**b,c**) Ey and Ez distribution along xy surface in the center of Si layer for the case TE(Ey dominant) is injected, and w_m_ is 140 nm (**b**) and 200 nm(**c). (d**–**f**) Polarization states on the Poincaré sphere of the output with finite long metal for rotating TE to (**d**) TM, (**e**) circular, and (**f**) linear 45° polarization. The red points on the Poincaré sphere stand for the position of the output polarization states.

The key to the waveguide design is that the top metal layer covers only a portion of the Si waveguide, thus breaking the horizontal symmetry and rotating the optical axis of original eigenmodes. The asymmetric HPW can support two orthogonal eigenmodes, and the mode optical axis angle θ, is defined as


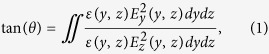


where ε(y, z) is the real part of the permittivity distribution, E_y_(y, z) and E_z_(y, z) are the transverse and horizontal electrical components of an eigenmode. Here the thickness of the thin SiO_2_ layer is 30 nm, which provides a good tradeoff between having relative low metal absorption loss and producing strong enough plasmonic effects to achieve 0° to 90° rotation in a short waveguide by properly designing the metal width, w_m_^17^. For example, Fig. 2(a) shows the magnetic field distribution of two eigenmodes for two typical cases when θ of the first mode is equal to either 45° or 22. 5°. The width of the metal waveguide coverage enables flexible tuning of the optical axis angle of the eigenmodes, which is an important waveplate parameter.

The other key parameter of a waveplate- the crystal thickness, corresponds to the metal length, L, in the waveguide device. L determines the phase difference of the two eigenmodes in the metal section. When light is injected from the input end and propagates to the metal section, the input mode decomposes into two eigenmodes of the asymmetric HPW, then the two modes interfere with each other and experience a phase difference at the end of metal due to their different phase velocities. The phase difference *δ* is


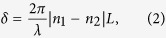


where n_1_ and n_2_ are the real part of effective indices of the two eigenmodes, and *λ* is the wavelength in free space. For example, when choosing a proper L to make *δ* equal to π or π/2, the device can work as a half waveplate or quarter waveplate, respectively. Consequently, we have two degrees of freedom in the waveguide design, the metal width and length, which produce an integrated, on-chip waveplate for any desired degree of polarization conversion.

3D FDTD simulations were first carried out to prove the concept. A TE mode wave was launched and three important cases were demonstrated—rotation to TM, circular and 45° linear polarizations. Based upon waveplate principles, for both rotation to TM and to circular polarizations, the eigenmode optical axis, θ, of the rotation section should be ± 45°, and the corresponding metal length providing *δ* to be π and π/2, respectively. Figure 2(b) shows the amplitude and phase changes of E_y_ and E_z_ without metal termination for θ is ± 45°. We can find that at L = 3 μm, E_y_ vanishes while E_z_ reaches a maximum, which means TE is rotated to TM; at L = 1.5 μm, both E_y_ and E_z_ are dominant components but with a π/2 phase difference, resulting a circular polarization. Polarization ellipse analysis of the two points where the metal lines terminate are shown in Fig. 2(d,e), which further confirm the expected results. In Fig. 2(d), the polarization is not exactly linear at 90° because the data is extracted from an output Si wire waveguide connected to the metal section, and the eigenmode of Si waveguides is not pure TE or TM but with small non-dominant electric components. In order to realize rotation from TE to linear 45° polarization, a “half-wave plate” is needed but with θ of 22.5°/−67.5°, based on the fact that half of the wave plate rotation between linear polarization states by an angle that is twice the difference between the input light angle and the optical axis of the waveplate. The 3D simulation corresponding to this is shown in Fig. 2(c). Similar to the circular polarization states, both E_y_ and E_z_ become dominant at half the beating length of around 3 μm, but differently, here E_y_ and E_z_ are in the same phase as marked by the red dashed line, resulting linear poalrization as shown in Fig. 2(f).

### Experimental demonstration

Experimentally, a series of devices with different metal lengths and widths were fabricated, together with Si waveguide references. The reference waveguides are exactly the same as the polarization rotators except that they have no metal coverage. For a fixed metal width, the output polarization states for various metal lengths from 0 (Si waveguide) to 4 μm were measured by a polarization measurement system.

**Figure 3:**
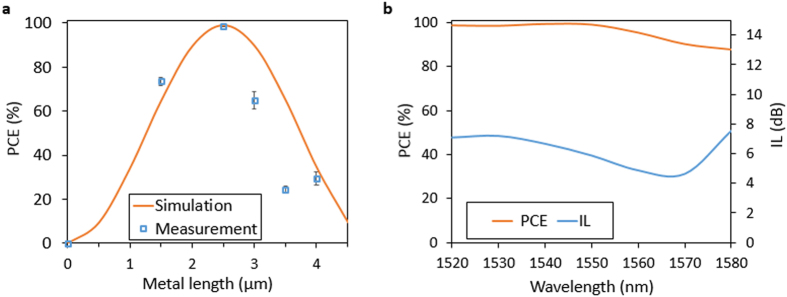
Measurement. (**a**) Measured and simulated polarization conversion efficiency (PCE) as a function of metal length at the wavelength of 1550 nm. The blue squares are average of measured results. Error bar is the standard deviation. (**b**) Measured spectrum response of PCE and insertion loss (IL) for the device with w_m_ = 140 nm and L = 2.5 μm.

Polarization conversion efficiency (PCE), defined as the output power ratio of the desired polarization to the sum of both polarizations, here PCE = P_TM_/(P_TE_ + P_TM_) for each metal length were calculated and compared to simulated results as shown in Fig. 3(a). The peak value of PCE is as high as 99.2% for a metal length of 2.5 μm at a wavelength of 1550 nm, and the insertion loss (IL) is 5.8 dB. Losses are mainly caused by these factors: absorption loss in metal, coupling losses (including mode mismatch and reflection) between the conversion segment and propagation loss from waveguide sidewall surface roughness. From simulations, the metal induced loss is around 1dB, which is relatively higher than previous theoretical work using Ag because in a practical fabrication process, a 2 nm Cr layer was used as an adhesion layer. Cr is much lossier than Ag and Au, and contributes substantially to the absorption loss, even though it is only 2 nm thick. The coupling loss between segments is around 1.2 dB. Thus overall losses can be further reduced by improved fabrication and better materials choices. Wavelength dependence of PCE and IL in the device with a metal length of 2.5 μm are presented in Fig. 3(b). PCE is above 91.5% for a wide band of 60 nm.

## Discussion

In summary, an ultracompact on chip waveplate is proposed and experimentally demonstrated with an extremely simple structure and high integration flexibility. Compared to previous polarization rotators which only convert between TE and TM modes, this device provides full functional polarization conversion from a linear input polarization to any angle of linear polarization or circular or elliptical polarization, by simply tuning the metal width and length, corresponding to the optical axis and crystal thickness of a bulk waveplate, respectively. Experimentally we demonstrate low loss, small footprint waveguide devices by rotating from TE to TM with a polarization conversion efficiency as high as 99.2% in a 2.5 μm long section. such a device paves the way to realize flexible polarization manipulation for various applications, such as coherent communications, bio sensing and quantum information processing that will significantly benefit from integration.

## Methods

### Fabrication

The device was fabricated using a SOI wafer. A thin SiO_2_ layer of 30 nm was created on top of the Si layer by high temperature (1000 °C) dry thermal oxidation. The waveguides were defined by electron-beam lithography (EBL) using negative maN-2405 resist. The pattern was transferred by dry etching, and the top SiO_2_ and Si layers were etched through to the buffer SiO_2_ layer using a plasma etcher. The pattern of the metal layer was defined by a second EBL writing using positive ZEP 520A resist. The resist thickness was over 200 nm to ensure good lift-off in the next steps. The metal layer (Au/Cr 60 nm/2 nm) was deposited by e-beam evaporation, and then lifted-off to form the metal strips that partially cover the Si waveguide. Finally the sample was clad with SiO_2_ using plasma-enhanced chemical vapor deposition.

### Numerical simulations

3D simulations of the device were performed using FDTD Solutions from Lumerical Inc. PML boundary conditions were applied for all boundaries. The input light was a mode source whose mode profile matched the TE eigenmode of the input Si wire waveguide.

### Measurement set-up

The sample was characterized using a waveguide-fiber alignment system. A tunable laser (wavelength ranging from 1520 to 1580 nm) was used as the light source. The output signal of the laser went through an in-line polarization controller (PC) and coupled to the device using a lensed fiber via butt coupling. At the output end, another lensed fiber was used to direct the light to a second PC and finally to a commercial polarization measurement system. The two PCs were calibrated using straight reference Si waveguides on the same chip. The measured extinction ratio and insertion loss were corrected by calibrating polarization dependent loss using a group of bare Si strip waveguides without metal strips.

## Additional Information

**How to cite this article**: Gao, L. *et al*. On-chip plasmonic waveguide optical waveplate. *Sci. Rep.* **5**, 15794; doi: 10.1038/srep15794 (2015).
